# Generalized myasthenia gravis with acetylcholine receptor antibodies: A guidance for treatment

**DOI:** 10.1111/ene.16229

**Published:** 2024-02-06

**Authors:** Nils Erik Gilhus, Henning Andersen, Linda Kahr Andersen, Marion Boldingh, Sini Laakso, Margret Oddny Leopoldsdottir, Sidsel Madsen, Fredrik Piehl, Trine Haug Popperud, Anna Rostedt Punga, Liselotte Schirakow, John Vissing

**Affiliations:** ^1^ Department of Neurology Haukeland University Hospital Bergen Norway; ^2^ Department of Clinical Medicine University of Bergen Bergen Norway; ^3^ Department of Neurology Aarhus University Hospital Aarhus Denmark; ^4^ Copenhagen Neuromuscular Center, Department of Neurology Copenhagen University Hospital Copenhagen Denmark; ^5^ Department of Neurology Oslo University Hospital Oslo Norway; ^6^ Department of Neurology, Brain Center Helsinki University Hospital Helsinki Finland; ^7^ Translational Immunology Research Program University of Helsinki Helsinki Finland; ^8^ MG Felag Islands Reykjavik Iceland; ^9^ The National Rehabilitation Center for Neuromuscular Diseases Aarhus Denmark; ^10^ Department of Clinical Neuroscience Karolinska Institutet Stockholm Sweden; ^11^ Department of Neurology Karolinska University Hospital Stockholm Sweden; ^12^ Department of Medical Sciences Uppsala University Uppsala Sweden; ^13^ Department of Clinical Neurophysiology Uppsala University Hospital Uppsala Sweden; ^14^ MG Group, The Danish Muscular Dystrophy Organization Aarhus Denmark

**Keywords:** acetylcholine receptor antibodies, immunosuppression, myasthenia gravis, thymus, treatment

## Abstract

**Background:**

Generalized myasthenia gravis (MG) with antibodies against the acetylcholine receptor is a chronic disease causing muscle weakness. Access to novel treatments warrants authoritative treatment recommendations. The Nordic countries have similar, comprehensive health systems, mandatory health registers, and extensive MG research.

**Methods:**

MG experts and patient representatives from the five Nordic countries formed a working group to prepare treatment guidance for MG based on a systematic literature search and consensus meetings.

**Results:**

Pyridostigmine represents the first‐line symptomatic treatment, while ambenonium and beta adrenergic agonists are second‐line options. Early thymectomy should be undertaken if a thymoma, and in non‐thymoma patients up to the age of 50–65 years if not obtaining remission on symptomatic treatment. Most patients need immunosuppressive drug treatment. Combining corticosteroids at the lowest possible dose with azathioprine is recommended, rituximab being an alternative first‐line option. Mycophenolate, methotrexate, and tacrolimus represent second‐line immunosuppression. Plasma exchange and intravenous immunoglobulin are used for myasthenic crises and acute exacerbations. Novel complement inhibitors and FcRn blockers are effective and fast‐acting treatments with promising safety profiles. Their use depends on local availability, refunding policies, and cost–benefit analyses. Adapted physical training is recommended. Planning of pregnancies with optimal treatment, information, and awareness of neonatal MG is necessary. Social support and adaptation of work and daily life activities are recommended.

**Conclusions:**

Successful treatment of MG rests on timely combination of different interventions. Due to spontaneous disease fluctuations, comorbidities, and changes in life conditions, regular long‐term specialized follow‐up is needed. Most patients do reasonably well but there is room for further improvement. Novel treatments are promising, though subject to restricted access due to costs.

## Graphical abstract

Myasthenia gravis experts and patient representatives from the five Nordic countries formed a working group to prepare treatment guidance based on a systematic literature search and consensus meetings. Successful treatment rests on a timely combination of symptomatic and immunosuppressive interventions. Due to spontaneous disease fluctuations, comorbidities, and changes in life conditions, regular long‐term specialized follow‐up is needed.

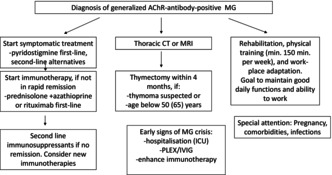

## INTRODUCTION

Myasthenia gravis (MG) is an autoimmune disorder that leads to muscle weakness due to antibodies against functionally important proteins in the postsynaptic membrane at the neuromuscular junction [1, 2]. Muscle weakness fluctuates over time and increases with repeated or sustained muscle use. Most patients have generalized weakness affecting the extraocular, bulbar, trunk, and extremity muscles. The weakness is more pronounced in proximal than distal muscles, and leg muscles are typically mildly weak or unaffected. The weakness is symmetrical, apart from the eyes. In 20% of MG patients, the muscle weakness remains confined to the extraocular muscles [3].

Most MG patients have pathogenic autoantibodies against the extracellular part of acetylcholine receptors (AChR), commonly associated with an abnormal thymus. Thymoma is present in 10% of such patients, and thymic hyperplasia with ectopic germinal centers appears in most patients with symptom onset before age 50 years [4]. Only a few percent of MG patients have antibodies against muscle‐specific tyrosine kinase (MuSK) and lipoprotein‐related protein 4 (LRP4) in the Nordic countries [5, 6]. In around 10% of patients with generalized MG, no antibodies are detected by standard testing. Many of these patients have low‐concentration or low‐affinity AChR antibodies when tested with more sensitive methods [7].

Patients with generalized MG need active and individualized treatment based on scientific evidence and approved guidelines. Major aspects to be considered are symptomatic drug therapy, thymectomy, immunoreactive drug treatment, physical training, and rehabilitation [8, 9]. Many patients do well with mild to minimal symptoms or even remission. However, 10%–20% of patients continue to have long‐standing moderate or severe weakness after treatment with at least two different immunosuppressive drugs at high doses [10]. Some 10% of MG patients experience a myasthenic crisis with respiratory muscle weakness and need of ventilatory support. MG patients have a slightly increased death rate, mostly in young females [11]. Even patients with only mild symptoms have a decreased quality of life because of fatigue, treatment side effects, and worries about exacerbations and complications [12, 13].

Well‐controlled and prospective studies for MG therapies are few, especially for long‐term follow‐up. Studies comparing alternative treatments are generally lacking. For some recent treatments, the effect in generalized MG is well documented, whereas several commonly used treatments lack such proof. Newer treatments are usually expensive, some drugs even extremely expensive, which calls for cost–benefit analyses. These are underway from the Nordic countries. Priorities, cost‐refunding policies, organization of health care, and accessibility of treatments vary among and within countries.

This article provides Nordic expert consensus treatment recommendations for generalized MG patients with AChR antibodies. It benefits from similar MG populations and health systems in Norway, Denmark, Sweden, Finland, and Iceland. Treatment and care are homogenous within the Nordic region and rely on government funding. Principles for treatment approval are laid down by governmental institutions, based on expert advice and decisions taken at the European level. Our author group combined MG experts and representatives from patient organizations. We believe our Nordic recommendations can be useful for MG treatment decisions worldwide.

## METHODS

In October 2022, the Danish Rehabilitation Center for Neuromuscular Diseases arranged a Nordic MG meeting with invited MG experts and patient representatives from Denmark, Norway, Sweden, Finland, and Iceland. The conclusion from the meeting was the need for updated and coherent MG treatment guidelines based on joint Nordic experiences. At a digital meeting, the working group for a guidance article was established in January 2023. The group met face to face in June 2023, several times virtually, and had extensive digital exchanges. Our recommendations are the result of consensus from these meetings. The working group members are identical to the list of authors. Since the members had complementary expertise and included patient representatives, we abstained from formal voting processes but instead obtained consensus through open discussions. The two patient representatives gave input on all aspects of the work, and particularly when deciding topics where MG‐specific treatment guidance was most needed [14].

All working group members wrote the first draft of specific sections of this article and/or gave input to all parts of the work. PubMed and Web of Science were searched by all authors of a first draft for the combination of “myasthenia gravis” and relevant co‐words. Hits were examined by title, relevant ones by abstract, and then by full text. Experimental studies were not included. Secondary articles were found from the reference lists of the examined articles. The selection of articles for examination and inclusion was based on relevance to this review's aims and scientific quality. We included only generalized MG with AChR antibodies, not ocular MG, MG with MUSK or LRP4 antibodies, or MG without detectable antibodies This MG group is the most frequent in the Nordic countries.

## RESULTS

### Symptomatic drug treatment

#### Acetylcholinesterase inhibitors

Acetylcholinesterase inhibitors are recommended as first‐line treatment in MG as they increase the amount of acetylcholine in the synaptic cleft, thus improving neuromuscular transmission [15, 16, 17]. Pyridostigmine bromide is the primary drug of choice [18]. Ambenonium chloride is usually less effective and is regarded as a second‐line acetylcholine esterase inhibitor [19].

Pyridostigmine dosage is titrated individually, where 30–60 mg every 4–6 h is a typical adult dose [20, 21]. In pediatric patients, titration starts from 1 mg/kg/day, and a typical dose is 7 mg/kg/day. A daily dose of 450–600 mg or more may cause increased muscle weakness and cholinergic crisis [15].

Side effects are mainly due to muscarinic adverse events, including stomach cramps, nausea, vomiting and diarrhea, muscle cramps, increased sweating, bronchial secretions, hypotension, and bradycardia [15]. In a Dutch–Belgian cohort, 91% reported side effects from pyridostigmine, and one in four discontinued the treatment mainly due to side effects [16]. Atropine in a low dose can be temporarily used to counteract such side effects, which are typically most pronounced at the start of the therapy. Pyridostigmine should not be given in the case of gastrointestinal or urinary obstruction and should be used cautiously in patients with bronchial asthma, bradyarrhythmias, or recent coronary occlusion. Pyridostigmine affects sensitivity to non‐depolarizing muscle relaxants such as vecuronium bromide [22].

#### Beta‐2 adrenergic agonists

Beta adrenergic agonists represent first‐line treatment for most congenital myasthenic syndromes but may also have a role in acquired MG. Beta‐2 adrenergic receptor agonists in low doses, mainly terbutaline, ameliorate the clinical symptoms in MG [23]. A randomized, placebo‐controlled, pilot study of terbutaline improved the quantitative MG score and the fatigue response on repetitive nerve stimulation [24]. Beta‐2 adrenergic agonists should be given with caution to patients with severe cardiovascular disease, uncontrolled hyperthyroidism, or hypokalemia.

#### Recommendations

- Pyridostigmine in optimal dose should be given as a first‐line symptomatic treatment in most patients with MG.
- The optimal dose can vary according to activity needs and be adjusted by the patient accordingly.
- Terbutaline and salbutamol can be used as adjuvant symptomatic treatments.

### Immunosuppressive drug treatment

#### Standard treatment

Immunotherapy should be offered to all patients who have not met their treatment goals despite optimal symptomatic treatment, and this accounts for more than 80% of patients. Immunosuppressive agents can be divided into corticosteroids and non‐steroidal immunosuppressants. Drugs used in MG include azathioprine, cyclosporine, mycophenolate mofetil, methotrexate, tacrolimus, and cyclophosphamide.

Early high‐dose oral prednisone or prednisolone is recommended (0.75–1.00 mg/kg) in patients with moderate to severe generalized MG [25]. In patients with mild to moderate generalized MG, steroids can be given at lower doses (0.25 mg/kg), followed by gradual escalation depending on the clinical response. Mild, moderate, and severe MG can be defined as MGFA class II–IV, but also from a reduction in patient‐reported activities of daily living, quality of life, and other MG scales [26]. Patient treatment needs should be individualized [13]. A gradual increase of steroid dose from 5 to 10 mg daily over a few weeks is recommended to avoid an initial worsening. If rapid response is needed, a high dose (30–50 mg daily) from the beginning can be combined with intravenous immunoglobulin or plasma exchange to avoid the steroid dip. In 75% of patients, steroids have an effect within 2–4 weeks. Once an acceptable MG status is obtained, the steroid dose should gradually, over months, be tapered to the lowest effective dose. In most patients, low‐dose steroids are well tolerated and provide good control of symptoms and deficits. Many centers prefer alternate‐day dosing of corticosteroids to minimize side effects. Good scientific evidence for a better outcome with this customary practice is lacking. Due to the high risk of long‐term adverse effects and to further improve muscle weakness, corticosteroids should be combined with another immunosuppressive drug. In some patients, corticosteroids can then be slowly withdrawn. Nordic countries tend to have a low chronic use of corticosteroids, only around 20% in a Danish cross‐sectional study [27], and around 30% in Norway 10 years ago [12].

MG patients with osteoporosis, obesity, diabetes, and mental disorders have a particularly high risk of corticosteroid side effects. Steroids should be given only for a short term for such patients in the lowest possible dose, and until another immunosuppressant has provided a good response. In children, short‐term treatment and low dose is particularly important. All patients on steroids should be monitored for blood pressure, body weight, bone mineral density, blood glucose levels, sleep, and mood problems. In patients with moderate or severe newly onset MG who need a fast response, short‐term immunomodulators such as intravenous immunoglobulin and plasma exchange should always be considered together with corticosteroids.

Azathioprine, mycophenolate, and methotrexate exert their effect by reducing T‐ and B‐cell proliferation, whereas the calcineurin inhibitors cyclosporine and tacrolimus primarily block T‐cell activation. These drugs have a delayed response of 1 to 6 months, and the full effect is sometimes reached after more than 1 year. They enable the tapering of steroids, improve the clinical response, and reduce the risk of steroid side effects. Non‐steroid immunosuppressants should be used alone if steroids are contraindicated or refused by the patient. In some patients it is possible to reduce steroids gradually until complete withdrawal and still maintain minimal symptoms or remission. Treatment should be initiated at a low or moderate dose. If tolerated, escalating doses should be given until the treatment targets are reached. Real‐life clinical experience demonstrates a positive effect in about 75% of patients. Azathioprine and cyclosporine have a proven effect in well‐controlled studies [28, 29]. For mycophenolate, methotrexate, and tacrolimus, the findings are conflicting [30], with some studies indicating a beneficial effect [31, 32]. In the Nordic countries, azathioprine (2–3 mg/kg) is usually the first‐line treatment in both adults and children. Mycophenolate (1–2 g daily), methotrexate (20 mg/week), and tacrolimus (3 mg daily) are secondary alternatives. The effect is dose‐dependent for all the drugs, and the dose should be increased if necessary to achieve an optimal clinical response. The activity of the enzyme thiopurine methyl transferase influences the effect of azathioprine and the risk of side effects and can be tested routinely. Azathioprine should be avoided in those with absent or low activity (<20 mU/L) [33]. Immunoglobulins, given intravenously or subcutaneously, are rarely used as maintenance therapy. A recent study found no steroid‐sparing effect of intravenous immunoglobulin [34]. Immunoglobulins are safe and have a temporary and fast‐acting effect, whereas long‐term effect data are insufficient [35, 36]. Number of immunoglobulin doses and dosing intervals should be individualized.

In most MG patients, there is a need to maintain immunosuppression for many years, sometimes lifelong. There is a lack of well‐controlled studies to determine when and how treatments can be tapered. Dose reduction of steroids and other immunosuppressants should always be considered in patients in long‐term stable remission or with only minimal symptoms, often after 1–2 years.

#### Recommendations

- In patients with MG‐related reduced quality of life or functional impairment despite optimal acetylcholinesterase inhibitor treatment, immunotherapy should be offered.
- Patients with moderate to severe generalized MG should be given high‐dose oral corticosteroids. In patients with mild generalized MG, corticosteroids can alternatively be given at lower doses, usually followed by gradual escalation.
- Patients with generalized MG should be given a non‐steroid immunosuppressant to achieve a better clinical response and enable steroid dose reduction.
- Azathioprine is recommended as the first‐choice non‐steroidal immunosuppressant drug.
- Mycophenolate, methotrexate, and tacrolimus are recommended as secondary immunosuppressants.
- Tapering of corticosteroids, and non‐steroidal immunosuppressants, should be considered after 1–2 years in patients in stable remission or with minimal symptoms.

#### Rituximab

Rituximab is a chimeric anti‐CD20 monoclonal antibody approved for B‐cell lymphoma, rheumatoid arthritis, and vasculitis. It eliminates immature, naïve, and memory B‐cells, but not plasma cells, from peripheral blood [37]. A meta‐analysis comprising 24 mostly retrospective studies across 417 patients with different forms of MG [38], the largest including 56 patients. [39], found a marked benefit of rituximab compared to control therapies [40]. Most used a weekly dose of 375 mg/m^2^ over 4 weeks and some used lower doses, with no dose‐dependent difference in effectiveness. Treatment benefits tended to be higher in mild to moderate than in severe MG. A more recent retrospective observational study included 77 AChR‐antibody‐positive patients with generalized MG, of whom 24 had new‐onset disease and 34 refractory symptoms. [41] Both groups displayed benefit, but this was greatest for newly onset MG.

Two randomized, placebo‐controlled studies with rituximab have been published. In BeatMG, rituximab 375 mg/m^2^ over 4 weeks was compared with placebo in 50 patients with generalized AChR‐antibody MG and ongoing immunotherapy, either only corticosteroids or in combination with other immunosuppressive therapies, assessing steroid‐sparing effect at 52 weeks and safety as co‐primary outcomes. [42] As 60% in the active arm and 56% in the placebo arm met the primary outcome efficacy criteria, results did not meet the predefined futility endpoint. In RINOMAX, 47 Swedish patients with new‐onset generalized MG (96% AChR‐antibody MG) were randomized to receive 500 mg rituximab or placebo as an add‐on to the standard of care. [43] Minimal disease manifestations at 16 weeks, defined as a Quantitative Myasthenia Gravis (QMG) score of 4 or less with a prednisolone dose ≤10 mg and no rescue treatment, was the primary endpoint. This was met by 71% in the active arm and 29% in the placebo group (*p* = 0.007). The same measure also favored rituximab at follow‐up until week 48.

In a meta‐analysis, rituximab was associated with adverse events among 20% of patients, most being mild to moderate but including one case of progressive multifocal leukoencephalopathy (PML) [38]. Exposure to rituximab over longer periods increases the risk of severe infections, but not of cancer [44, 45]. There are no randomized studies of rituximab in children with MG, but the drug should be considered if moderate and severe symptoms [46], especially if newly onset.

Use of rituximab for non‐MG indications shows that some patients develop hypogammaglobulinemia that may take a long time to reverse. We recommend measuring immunoglobulin G (IgG) before each rituximab dose and extend intervals or stop treatment with decreasing concentrations. There is only limited evidence to suggest that measurement of CD19+ total B‐cells is useful in deciding rituximab dosing intervals. However, a return of CD19+ CD27+ memory B‐cells may predict risk of MG worsening [47].

#### Recommendations

- Rituximab should be considered a first‐line immunosuppressive drug in newly onset disease, as an alternative to steroids and azathioprine.
- Rituximab should be considered for treatment‐refractory symptoms, although evidence for its effectiveness is weaker than in newly onset disease.
- There is no clear benefit with single doses exceeding 500 mg, or with repeated pulsed doses.
- A second dose should be considered after 6 months or longer, depending on clinical response.
- Serum IgG levels should be measured before each re‐dosing.
- Determination of B‐cell populations may be used as a tool to individualize dosing intervals.

### Complement inhibitors and FcRn blockers

Several inhibitors of complement proteins have appeared within the last 5 years as a new treatment modality for refractory or difficult‐to‐treat MG patients [48, 49, 50, 51, 52, 53]. Such drugs are eculizumab, ravulizumab, and zilucoplan, with several more in ongoing trials. The action of this downstream target of the immune cascade preserves immunoglobulins and B‐ and T‐cells. Complement plays a major role in the destruction of the postsynaptic membrane in MG with AChR antibodies. The treatment effect is seen within the first 2 weeks and maximal effect appears within the first 2 months. More than half of the patients with chronic disease seem to respond even as a second‐ or third‐line therapy, and with a clinically meaningful improvement. Safety has been shown to be acceptable. Limitations include the need for meningococcal vaccination before starting treatment, pneumococcal vaccination which is recommended, and regular intravenous or subcutaneous injections. Prophylactic antibiotic treatment is not recommended for most patients. Very high drug costs have limited the accessibility to treatment in most countries. Formal approval, guidelines for practical use, and refund policies vary between and sometimes within countries. Inhibitors of other complement protein targets, as well as alternative modes of drug administration, are being developed.

The FcRn receptor is responsible for the recycling of proteins across cell membranes. Proteins bind to the FcRn receptor during endocytosis and therefore avoid destruction via lysosomal breakdown. Monoclonal antibodies directed at the FcRn receptor's binding site for IgG block recycling and lead to a 60%–70% reduction in circulating IgG. Elimination is selective for IgG, has no direct effect on cellular immune responses, but has no specificity for the pathogenic MG autoantibodies. The therapeutic effect appears within the first 2 weeks and is obtained to the same degree with repeated cycles of treatment [53, 54, 55, 56]. The most prominent side effect is headache, which usually recedes on continued treatment. The treatment can be tailored according to the disease state; new treatment cycles can be administered with signs of symptom worsening or according to a fixed schedule. The number of treatment responders and the magnitude of the treatment response for FcRn blockers and complement inhibitors seem to be similar. No prospective and controlled head‐to‐head studies have been undertaken. Both complement inhibitors and FcRn blockers may also be a treatment alternative during acute and severe exacerbations. FcRn blockers inhibit the transfer of IgG across the placenta during pregnancy, which may be relevant for women with MG and the risk of neonatal MG, spontaneous abortion, and arthrogryposis [57]. One FcRn blocker, efgartigimod, is licensed and available in some countries and several other FcRn blockers are on the way to being marketed, including rozanolixizumab, or under investigation, such as batoclimab and nipocalimab. In common with complement inhibitors, the availability of FcRn blockers is limited by high drug costs.

#### Recommendations

- The use of complement and FcRn inhibitors depends on local availability, formal approval, and refund policies.
- Due to high drug costs, complement and FcRn inhibitors are recommended only for difficult‐to‐treat patients with severe MG not responding to standard immunosuppressive treatment.
- Complement and FcRn inhibitors act fast, have a proven effect with an acceptable safety profile, with a selective impact on the immune system. If costs permit and are acceptable, they should be recommended as alternative second‐line treatments.
- Complement and FcRn inhibitors are recommended as second‐ or third‐line treatment in severe MG exacerbations and crisis.

### Thymectomy

A computed tomography (CT) or magnetic resonance imaging (MRI) scan should be performed in all patients with newly diagnosed MG to identify thymoma and thymic hyperplasia, though sensitivity for hyperplasia is very low. In MG patients with a thymoma, thymectomy represents a key treatment to improve survival, and also improves MG symptoms in most patients, with complete stable remission reported in up to 30% [58]. This treatment should be performed in specialized centers covering thoracic surgery, neurology, and oncology for further treatment and follow‐up.

Thymectomy improves symptom management and lessens the need for immunosuppressive medication in patients with generalized MG, AChR antibodies, and those aged 18–50 years. This has been shown in a randomized controlled trial including patients under 65 years with MG for less than 5 years [59]. Primary outcome measures were time‐weighted average quantitative MG score and time‐weighted average alternate‐day prednisone dose. Secondary outcome measures were azathioprine use, intravenous immunoglobulin use, and hospitalizations for MG exacerbations. Both primary and secondary outcomes were better for the thymectomized patients, and for patients both under and over 40 years of age. Subgroup analysis of individuals above age 50 years was inconclusive. There was a continued benefit of thymectomy after 60 months [60]. Thymic pathology (i.e., number of germinal centers) correlated with a better outcome after thymectomy [61]. In a systematic review and meta‐analysis of late‐onset MG, thymectomy was not superior to conservative treatment for complete stable remission or pharmacological remission [62]. Several retrospective studies indicate an effect of thymectomy up to age 65 years. Thymectomy is also recommended in children [62, 63].

In most centers, thymectomy is performed using video‐ or robot‐assisted thoracic surgery. Complete removal of all thymic tissue is important to obtain the full effect of thymectomy. There seems to be a similar outcome with the minimally invasive techniques and transsternal thymectomy [64, 65, 66]. Minimally invasive techniques are preferred with fewer perioperative complications, shorter hospitalization time, and minimal scars.

#### Recommendations

- A thoracic CT or MRI scan should be performed at the time of MG diagnosis to examine for thymoma.
- Thymomas should be surgically removed due to the risk of local invasion.
- In patients with non‐thymoma, generalized MG, AChR antibodies, and age below 50 years, thymectomy should be performed within 4 months from diagnosis. The patient should be in a stable clinical condition.
- In patients with non‐thymoma generalized MG, AChR antibodies, and age 50–65 years, early thymectomy should be considered if minimal manifestations are not reached, or the patient has substantial side effects from treatment. Biological age, comorbidities, patient preference, and other individual factors should be considered.
- Minimally invasive surgery is recommended. Special attention should be taken to remove all thymic tissue.

### New and experimental treatment

Several novel drugs are in clinical development for MG [67, 68]. Based on mode of action, they can be classified into those that deplete selected cell populations and those that modulate immune signaling or immune cell activation. The anti‐CD19 monoclonal inebilizumab, recently approved for neuromyelitis optica, is in phase 3 clinical testing for generalized MuSK MG and AChR MG (NCT04524273). CD19 expression in the B‐cell lineage partly overlaps with CD20, but since CD19 expression remains for longer in plasma cell differentiation, the treatment effect on antibody production can be expected to be greater. TAK‐079 is an anti‐CD38 monoclonal that depletes plasma cells. A phase 2 trial (NCT04159805) in generalized MG indicated no effect from baseline up to week 32, although autoantibody levels tended to drop more with active treatment. In a single case report, the anti‐CD38 daratumumab gave a substantial clinical improvement in severe MG. [69] Cladribine is an oral purine analogue that selectively depletes lymphocytes. In a case series comprising 13 patients with treatment‐refractory MG, 11 experienced clinical improvement [70]. A phase 2 trial with chimeric antigen receptor (CAR) T‐cells recognizing the B‐cell maturation antigen (BCMA) in generalized MG is ongoing (NCT04146051). Several case reports and case series with clinical improvement following autologous hematopoietic stem cell transplantation for refractory MG have been published [71].

Modulation of interleukin‐6 (IL‐6) signaling affects the production of autoantibodies and suppresses disease activity in neuromyelitis optica [72], and a phase 3 trial with satralizumab in generalized MG is ongoing (NCT04963270). Tocilizumab is approved for rheumatoid arthritis and modulates IL‐6 signaling. Clinical improvement in two MG patients with refractory symptoms has been published [73], and a phase 2 trial is ongoing (NCT05067348). Telitacicept is a fully human TACI‐Fc fusion protein targeting B lymphocyte stimulator (BLyS) and a proliferating‐inducing ligand (APRIL) in late‐stage development for systemic lupus erythematosus, with a phase 3 trial in generalized MG ongoing (NCT05737160).

#### Recommendations

- None of the listed therapies have robust evidence showing clinical benefit in MG. They may be considered only in highly selected, refractory, and very severe cases with unsatisfactory effect or intolerability to standard therapies.
- Tocilizumab has a good tolerability profile and may be considered in MG with severe or moderately severe refractory symptoms.
- Cladribine and inebilizumab may be considered in severe, refractory MG.
- Stem cell transplantation is recommended only for the most severe treatment‐refractory MG, and only in centers with low complication rates.

### MG crisis

Myasthenic crisis is a serious exacerbation of MG, where ventilatory or bulbar dysfunction causes an imminent threat of death due to respiratory failure, and intubation or non‐invasive ventilation is needed [17]. A retrospective study of 815 patients found severe MG at diagnosis, thymoma, and MuSK antibodies to be risk factors [74]. In a study of 250 MG patients requiring mechanical ventilation, advanced age, onset after age 50 years, severe MG at debut, and comorbidities increased the risk for need of mechanical ventilation for over 15 days [75]. Myasthenic crisis occurs in around 10% of patients with generalized MG within the first 2 years from diagnosis [76], with a mortality rate of 5%–10% [75, 76, 77]. The respiratory capacity can drop quickly and continuous observation in hospital is necessary during severe MG exacerbations. Vital capacity and blood gas examinations do not reliably predict the need for respiratory support.

Immediate and optimal management of the airways with respiratory and circulatory support is essential [78]. Pyridostigmine should be discontinued during a crisis to prevent increased mucus secretion and plugging of the airways, and restarted before weaning from mechanical ventilation [78].

The two first‐line treatments, plasma exchange and intravenous immunoglobulin, have similar efficacy according to two well‐controlled trials [79, 80]. A systematic review and meta‐analysis indicated a slightly higher response rate and faster clinical improvement with plasma exchange [78, 81]. An experienced team reduces side effects and complications [82]. When a prolonged and severe crisis is expected, a sequential combination of both treatments is superior, with plasma exchange being given first so as not to wash out the immunoglobulins [75, 78]. Intravenous immunoglobulin dose is usually 2 g/kg body weight as a total dose given over 3–5 consecutive days. Selective plasma exchange with specific immunoadsorption is possible but is not widely used and does not have a better effect [83]. Very high doses of corticosteroids can be used as add‐on therapy in intensive care (prednisone 3–4 mg/kg or parenteral methylprednisolone 500 mg per day) [84]. This treatment is recommended in case of a delayed (>1 week) or insufficient response to the primary therapy.

#### Recommendations

- Early signs of a myasthenic crisis should lead to immediate intensive care unit admission.
- Plasma exchange and intravenous immunoglobulin are the primary treatments recommended for crisis. The choice should be made based on availability and individual patient assessment.
- After a myasthenic crisis, the immunotherapy should usually be intensified with increased drug doses and/or additional drugs.

### Physical activity and training

The World Health Organization (WHO) recommends that adults undertake regular physical activity for at least 150 min per week at moderate or 75 min per week at vigorous intensity. There has been a fear of overuse muscle damage in MG patients.

An interventional study found improvement in muscle strength in 11 patients with MG during a supervised 10‐week resistance training period [85]. Further studies on supervised aerobic and/or resistance training for 8–24 weeks found that exercise in MG was safe, well‐tolerated, and positively influenced muscle strength and performance‐based outcome measures [86, 87, 88, 89, 90]. Rahbek et al. [86] included 15 mildly to moderately affected patients with generalized MG for an 8‐week training program, including 20 training sessions with either progressive resistance training or aerobic training. The progressive resistance group showed increased maximal strength and functional capacity. A recent study found that exercise was safe but with no improved health‐related quality of life [89]. An open‐label case study found that individually tailored exercise programs under the guidance of a personal trainer were safe and improved aerobic capacity, muscle strength, and balance [91].

Studies have examined habitual physical activity in patients by questionnaires [92], accelerometer [93, 94], or in combination [95]. MG patients were less active than controls and did not meet the WHO recommendations for physical activity. An association between moderate or high levels of physical activity and low levels of fatigue was found in a cross‐sectional study of 779 Danish MG patients [92]. Similarly, lower fatigue scores were found in physically active Dutch patients [96]. Only minor and unsustained improvement in fatigue occurred after a 10‐week program with physical activity, relaxation, patient education, and psychological interventions [97]. There was no improvement in fatigue after 8 weeks of aerobic and resistance training [86].

Some MG patients feel exhausted after physical activity. This tends to improve after the first few weeks of exercise. Focused training secures a balance between exercise and fatigue. Patients often prefer to plan their physical activity in accordance with their pyridostigmine intake.

In patients with an MG crisis or at risk of developing a crisis, heavy physical activity and training should be avoided.

#### Recommendations

- Physical activity is safe and beneficial for most patients with MG.
- Training and exercise improve muscle strength and functional outcomes in MG.
- Physical activity for at least 150 min per week at moderate intensity is recommended for stable MG patients.
- MG patients benefit from individualized exercise programs supervised by a physiotherapist or in a gym.

### Rehabilitation and work

MG patients need to ration their daily activities, impacting their ability to combine full‐time jobs, perform tasks at home, and participate in standard social activities. A Danish nationwide cohort study found that MG patients had almost six times higher odds of no labor market participation and long sick leave 2 years after diagnosis than the general Danish population [98]. An Australian study among 165 MG patients found that 40% had stopped working and 20% had changed their occupation due to MG [99]. In a qualitative study from the United States, all 28 MG participants reported impacts on their working activities and financial situation [100].

We recommend that rehabilitation takes place in a multi‐professional network and that the healthcare professionals are aware of and address the challenge of finding the right work–life balance from an early stage. Since MG is a chronic disease, this must be addressed throughout life.

In the time from diagnosis or worsening of the MG symptoms until the full effect of treatment, MG patients may find themselves with the uncertainty of not knowing their new level of function and often at the same time feeling pressure from their employer. The unpredictability and fluctuating nature of MG remains both a physical and psychological challenge [101]. Psychological intervention and experienced peers sharing coping skills, combined with optimal treatment and precise, individualized information from healthcare professionals, are needed and should increase the level of function.

#### Recommendations

- The aim of rehabilitation and treatment should be to enable most MG patients to continue professional work, either part‐time or full‐time.
- Coordinated rehabilitation, including dialogue with the workplace, insurance companies, and social services, is needed. Due to physical impairment, fatigue, and possible treatment side effects, work adaptations are often necessary.
- Psychological intervention, including experienced peers sharing coping skills, can increase the patient's level of function. Psychological support and referral to an experienced psychologist should be offered.
- Information about relevant patient advocacy groups should be given, as such groups can help with coping and information.

### Special attention groups

#### Pregnancy and breastfeeding

Most women with MG of childbearing age require immunosuppressive drugs [102]. There is an increased risk of MG worsening during pregnancy [103]. Thymectomy should be performed before pregnancy as it reduces the risk of neonatal MG [104, 105]. An important question is whether MG drugs have teratogenic effects on the unborn child and during lactation. Recommendations are based mainly on registry studies, long‐term observation studies, and experience from treating other and more common diseases [57, 106, 107, 108].

Pyridostigmine is safe during conception and pregnancy. Emesis and changes in absorption during pregnancy may necessitate dose adjustment. Corticosteroids in low doses and azathioprine are regarded as safe and can be continued during pregnancy and breastfeeding [109]. Daily doses should be as low as possible, and prednisone/prednisolone below 20 mg is recommended [109, 110]. Rituximab should, if possible, be discontinued 3 months before pregnancy, or alternatively from conception [57]. Mycophenolate, methotrexate, and cyclophosphamide should not be used in women of childbearing age [17, 107]. Tacrolimus and cyclosporin are regarded as having very low or no increased risk but should be used in the lowest effective dose. Intravenous immunoglobulin and plasma exchange are recommended as rescue therapy during pregnancy and in the postpartum period [109]. Complement inhibitors and FcRn blockers have limited data to support safety in pregnancy and are not recommended. The FcRn blockers inhibit IgG transport from mother to child and may become a treatment alternative during the first two trimesters to avoid fetal AChR inactivation syndrome and arthrogryposis in the child [109].

Women with MG should give birth in hospitals with a neonatal intensive care unit. Vaginal delivery is recommended, and caesarean section should be advised for obstetric indications only [57, 107]. Epidural analgesia is preferred [111]. The use of magnesium should be avoided as treatment for pre‐eclampsia and eclampsia [108].

Immunosuppressive drugs should be restarted postpartum if stopped before pregnancy because of increased MG exacerbation risk in this period. Breastfeeding is recommended, but not for mycophenolate, methotrexate, and cyclophosphamide users [109]. Monoclonal antibody transfer is low in breast milk and peaks within 48 h [112]. Rituximab is recommended from 2 weeks after birth, with breastfeeding from 6 h after infusion.

#### COVID and vaccination

The risk of severe COVID‐19 infection is increased in MG [113]. This risk increases with age and decreases with vaccination [114]. COVID‐19 and influenza vaccinations are recommended in MG, and patients with a reduced immune response due to high‐dose immunosuppressive treatment should have repeated vaccinations [115]. They obtain a humoral immune response, but this is somewhat delayed [116]. MG patients are good candidates for antiviral treatments to protect from MG exacerbations [117].

#### Recommendations

- Thymectomy should be performed before pregnancy.
- Pyridostigmine, corticosteroids in daily doses below 20 mg, and azathioprine are considered safe during pregnancy and breastfeeding.
- Rituximab can be used until 3 months before conception and during breastfeeding.
- Intravenous immunoglobulin and plasma exchange are safe treatments during pregnancy and postpartum.
- Regular follow‐up by a neurologist and an obstetrician is recommended during pregnancy.
- Women with MG should give birth in a hospital with neonatal intensive care unit.
- Vaginal birth is recommended, and caesarean section should be performed by the same obstetric indications as for the general population.
- Breastfeeding is generally recommended.
- Vaccinations are recommended according to standard programs.

## DISCUSSION

**FIGURE 1:**
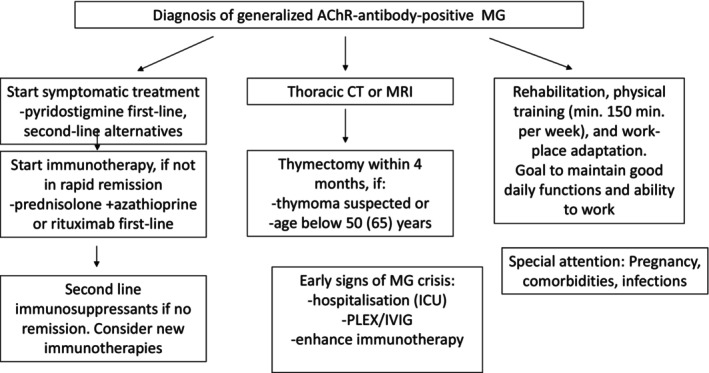
Schematic presentation of treatment and follow‐up considerations for patients with generalized myasthenia gravis (MG) and antibodies against the acetylcholine receptor (AChR) in skeletal muscle. CT, computed tomography; ICU, intensive care unit; IVIG, intravenous immunoglobulin; PLEX, plasma exchange; MRI, magnetic resonance imaging.

Treatment of patients with generalized MG and AChR antibodies should rely on evidence from controlled studies. However, such evidence needs to be complemented with real‐world data. Personalized medicine implies individual assessments of the benefit–risk balance and, equally important, engaging the patient in treatment decisions. Our guidance paper combines the current evidence base with clinical empirical experience, including that of user representatives, to devise Nordic consensus recommendations. These recommendations consider the effectiveness and safety of interventions in the short‐ and long‐term perspective and attempt a holistic approach. Increased infection risk is a concern for all types of immunosuppression [118]. The lowest possible drug dose and relevant vaccinations are recommended. A summary of our recommendations is illustrated in Figure 1.

Optimization of therapy along the disease course requires regular specialist follow‐up. Far from all treatments are available worldwide. Novel and very expensive MG treatments are restricted even in high‐income countries. The prognosis of MG has improved, but a combination of access to MG expertise and the entire treatment arsenal will be needed to further improve long‐term outcomes. Patients and doctors should together define ambitious treatment goals [13].

Recently approved therapies comprising complement and FcRn inhibitors display efficacy with rapid onset in most patients and good tolerability in well‐controlled trials. Real‐world experience is still limited but corroborates trial data. Availability and refund policies vary between and within countries, mainly because of the very high costs.

## CONCLUSIONS

First‐line symptomatic therapies for generalized MG with AChR antibodies comprise pyridostigmine, adapted and regular physical training, and social rehabilitation and support when needed. Immunosuppressive treatment should be given to most patients. Early thymectomy, prednisolone, azathioprine, and rituximab all represent first‐line therapies. In most patients several treatments are combined. Second‐line treatments are mycophenolate, methotrexate, and tacrolimus. Complement inhibitors and FcRn blockers are effective treatments with a rapid onset of action, but their availability as second‐line treatments is still limited for health economic reasons.

First‐line treatments for acute exacerbations and MG crisis are plasma exchange and intravenous immunoglobulin, combined with careful observation at an adequate care level, including intensive care. High‐dose corticosteroids, FcRn blockers, and complement inhibitors represent second‐line treatments.

Treatment of MG should always include a combination of interventions. From our Nordic experience, we recommend devising an individualized MG treatment strategy at a highly specialized unit with experience and updated knowledge. Active user involvement and an individualized user perspective are mandatory. Regular quality assessments of ongoing treatment are recommended, and should consider updated local, national, and international guidelines and recommendations.

## AUTHOR CONTRIBUTIONS

**Nils Erik Gilhus:** Conceptualization; investigation; funding acquisition; writing – original draft; methodology; validation; visualization; writing – review and editing; project administration; supervision; resources. **Henning Andersen:** Conceptualization; investigation; methodology; validation; writing – original draft; writing – review and editing. **Linda Kahr Andersen:** Conceptualization; investigation; writing – original draft; methodology; validation; writing – review and editing. **Marion Boldingh:** Conceptualization; investigation; writing – original draft; writing – review and editing; methodology; validation. **Sini Laakso:** Conceptualization; investigation; writing – original draft; methodology; validation; writing – review and editing; visualization. **Margret Oddny Leopoldsdottir:** Conceptualization; writing – review and editing; methodology; validation. **Sidsel Madsen:** Conceptualization; investigation; writing – original draft; methodology; validation; writing – review and editing. **Fredrik Piehl:** Conceptualization; investigation; writing – original draft; methodology; validation; writing – review and editing; supervision. **Trine Haug Popperud:** Conceptualization; investigation; writing – original draft; methodology; validation; writing – review and editing. **Anna Rostedt Punga:** Conceptualization; investigation; writing – original draft; methodology; validation; writing – review and editing. **Liselotte Schirakow:** Conceptualization; investigation; funding acquisition; methodology; validation; writing – review and editing. **John Vissing:** Conceptualization; investigation; methodology; validation; writing – review and editing.

## CONFLICT OF INTEREST STATEMENT

N.E.G. has received financial support from UCB, Argenx, Janssen, Merck, Roche, Alexion, Immunovant, Octapharma, Huma, Denka, Grifols, and Dianthus. H.A. has received financial support from UCB, Argenx, Roche, Horizon Therapeutics, Lundbeck, Novo, Alexion, Sanofi‐Genzyme, NMD Pharma, and Octapharma. M.B. has received consultant fees from Argenx and as an investigator for UCB. S.L. has received lecture fees from Argenx, Biogen, Janssen, Merck, Novartis, Roche; congress expenses from Merck, Novartis; advisory fees from Argenx, Novartis, Roche, Sanofi, and UCB; and serves as an investigator for the clinical study Clarion (Merck) and subinvestigator for the clinical study Fenhance (Roche). F.P. has received research grants from Janssen, Merck KGaA, and UCB, and fees for serving on data monitoring committees in clinical trials with Chugai, Lundbeck, and Roche, and preparation of expert witness statement for Novartis. T.H.P. has received a research grant from Octapharma and speaker's honoraria from Argenx and Alexion. A.R.P. has received consultancy fees from Argenx, UCB, Dianthus, and Toleranzia. L.K.A., M.O.L., S.M., and L.S. report no conflicts of interest. J.V. has been a paid consultant for UCB, Horizon Therapeutics, Argenx, Janssen, Roche, Alexion, Immunovant, and Dianthus, and is the Principal Investigator on clinical trials sponsored by UCB, Argenx, Janssen, Roche, Alexion, Horizon Therapeutics, and Regeneron. The other authors have no conflicts of interest.

## Data Availability

Data sharing is not applicable to this article as no datasets were generated or analyzed during the current study.
